# Systematic induced resistance in *Solanum lycopersicum* (L.) against vascular wilt pathogen (*Fusarium oxysporum f*. *sp*. *lycopersici*) by *Citrullus colocynthis* and *Trichoderma viride*

**DOI:** 10.1371/journal.pone.0278616

**Published:** 2023-05-02

**Authors:** Athirstam Ponsankar, Sengottayan Senthil-Nathan, Prabhakaran Vasantha-Srinivasan, Raghuraman Pandiyan, Sengodan Karthi, Kandaswamy Kalaivani, Muthiah Chellappandian, Radhakrishnan Narayanaswamy, Annamalai Thanigaivel, Krutmuang Patcharin, Shahid Mahboob, Khalid Abdullah Al-Ghanim

**Affiliations:** 1 Division of Bio-pesticides and Environmental Toxicology, Sri Paramakalyani Centre for Excellence in Environmental Sciences, Manonmaniam Sundaranar University, Alwarkurichi, Tirunelveli, Tamil Nadu, India; 2 Department of Bioinformatics, Saveetha Institute of Medical and Technical Sciences (SIMATS), Saveetha School of Engineering, Chennai, India; 3 Department of Entomology, University of Kentucky, Lexington, Kentucky, United States of America; 4 Post Graduate and Research Department of Zoology, Sri Parasakthi College for Women, Courtrallam, Tirunelveli, Tamil Nadu, India; 5 PG and Research Department of Botany, V.O. Chidambaram College, Thoothukudi, Tamil Nadu, India; 6 Department of Biochemistry, Saveetha Institute of Medical and Technical Sciences (SIMATS), Saveetha Medical College and Hospital, Chennai, Tamil Nadu, India; 7 Faculty of Agriculture, Department of Entomology and Plant Pathology, Chiang Mai University, Chiang Mai, Thailand; 8 Faculty of Agriculture, Innovative Agriculture Research Center, Chiang Mai University, Chiang Mai, Thailand; 9 Department of Zoology, College of Science, King Saud University, Riyadh, Saudi Arabia; Chandigarh University, INDIA

## Abstract

The antifungal effects of *Citrullus colocynthis* extract (Hexane, chloroform, methanol, and water) were tested *in vitro* on *Fusarium oxysporum* f. sp. *lycopersici* (Sacc.) W. C. Snyder & H. N. Hans (*FOL*), the causal agent of Fusarium wilt. Of these, methanol and water extract at 10% showed the highest inhibition of mycelial growth of *FOL* by 12.32 and 23.61 mm respectively. The antifungal compounds were identified through Fourier transform infrared (FT-IR) spectroscopy and gas chromatography-mass spectroscopy (GC-MS). The methanol extract was compatible with the biocontrol agent *Trichoderma viride*. The antagonistic fungi were mass-cultured under laboratory conditions using sorghum seeds. Both *T*. *viride* and *C*. *colocynthis* methanol extract was also tested alone and together against *FOL* under both *in vitro* and *in vivo* conditions. The combination of *T*. *viride* and *C*. *colocynthis* showed the highest percentage of antifungal activity (82.92%) against *FOL* under *in vitro* conditions. This study revealed that induced systemic resistance (ISR) in enhancing the disease resistance in tomato plants against Fusarium wilt disease. The combined treatment of *T*. *viride* and *C*. *colocynthis* significantly reduced the disease incidence and index by 21.92 and 27.02% in greenhouse conditions, respectively. Further, the induction of defense enzymes, such as peroxidase (PO), polyphenol oxidase (PPO), β-1,3-glucanase, and chitinase were studied. The accumulation of defense enzyme was greater in plants treated with a combination of *T*. *viride* and *C*. *colocynthis* compared to the control. Reduction of wilt disease in tomato plants due to the involvement of defense-related enzymes is presumed through this experiment.

## 1 Introduction

Tomato (*Solanum lycopersicum*) (L.) is a primary vegetable crop, extensively cultivated throughout the world, especially in European Union countries, China, USA, India, and Turkey [1,2]. More than 65% of nations have expanded processing tomato production because of the specific nutritional assessment [3,4]. In India, the tomato was cultivated on 290.3 thousand ha of the land area during 1990 which grew to 479.2 thousand ha with a production level of 17.91Mgha−1 [5,6]. Though this is an important commercial crop, production levels are hampered by several pathogens and pests [7,8].

An estimated 37% of crop loss is due to pests, of which 12% is to pathogens [9]. The most devastating plant pathogens belong to fungi in the genus *Fusarium* and these result in hundreds of millions of dollars in lost profits annually [10]. Solanaceous crops (tomato, pepper, eggplant, and potato) are susceptible to infection by *Fusarium* at all ages resulting in Fusarium wilt. In tomato crops the pathogenic form of *Fusarium oxysporum* f. sp. *lycopersici* (Sacc.) W. C. Snyder & H. N. Hans (*FOL*) causes classic vascular wilt disease, which blocks the xylem resulting in water blockage. A major problem with the fungus *FOL* is that it survives for long periods, up to 30 years in soil, removing land from the production of any susceptible agricultural crops [11,12]. The phytopathogen *FOL* is cosmopolitan and has spread worldwide causing great concerns in food and economic security.

Chemical fungicides are generally not effective in the control of Fusarium wilt [13]. Overuse of fungicides has resulted in an imbalance in the soil microbial community affecting the beneficial microbes and resulting in pathogen resistance. To overcome these problems, resistant varieties or grafting the susceptible cultivars onto resistant rootstocks generally followed farmers. In many developing countries grafting is not economically feasible. Proposed biological control methods provide one solution which may be effective and affordable [14]. However, dependence upon a single biocontrol agent often results in inconsistent field performance [8]. Alternative approaches using biocontrol organisms now promote using multiple agents in combination with plant improvements or commercial products to provide better control measures [15].

Naturally, plants possess sophisticated defense strategies to secure themselves from the attack of phytopathogens [16]. Systemic acquired resistance (SAR) and induced systemic resistance (ISR) are two forms of induced resistance wherein plant defenses are preconditioned by prior infection or treatment that results in resistance against subsequent challenge by a pathogen or parasite [17]. Induced systemic resistance (ISR) triggers multiple defense mechanisms including amplified activity of pathogenesis-related (PR) proteins like chitinase, β-1,3-glucanase and peroxidase (PO), and also the polyphenol oxidase (PPO). Chitinases and β-1,3-glucanases are hydrolytic enzymes involved in the plant’s defense against phytopathogens [18]. These enzymes degrade the cell wall of fungi by secreting lysis enzymes (Chitin and glucan oligomers) which perform as an elicitor and prevent the plant from pathogen attack. The enzymes PO are involved in the metabolism of phenyl propanoid [19], while the PPO catalyzes the oxidation of phenolic compounds to toxic quinones which have an important role in plant pathogen resistance [20,21].

*Trichoderma* species are a beneficial cosmopolitan group of fungi that are predominant in soils across different ecosystems [22]. In addition to colonizing roots, these fungi attack, parasitize and suppress other pathogenic fungi [23,24]. *Trichoderma* species can withstand adverse environmental conditions and reproduce rapidly, hence they are highly aggressive phytopathogens [25]. Mycoparasitism is not the only mode of action but it is one of the biocontrol mechanisms deployed by Trichoderma. Trichoderma deploys different biocontrol mechanisms, which could be direct (enzymes, mycoparasitism, fungicides, bioactive and even volatile compounds) and indirect (competition, rhizosphere competence, and priming of plant immunity) [24,26,27].

The Discovery and use of novel phytochemicals have emerged as an important resource of new agrochemicals for the control of many types of plant pathogens. For example, the Cucurbitaceae family consists of herbaceous climbers of annual plants with over nine hundred species [28]. *Citrullus colocynthis* (L.) Schrad, of this family, is broadly distributed around the warmer areas of the world including West Asia and Tropical Africa and is widely distributed in India, Pakistan, and Saudi Arabia [29,30]. *C*. *colocynthis* fruits are commonly known as ground melons [29,31]. Phytochemicals from the fruits, roots, and leaves of *C*. *colocynthis*, were shown to have antibacterial activity substances from aqueous and hydromethanolic extracts of leaves, roots, and fruits against *Klebsiella pneumonia*, *Bacillus stearothermophilus*, and *Staphylococcus aureus* [32].

This work investigates the antimicrobial activity of the fungal biocontrol agent *T*. *viride* with extracts of fruits from *C*. *colocynthis* against the severe fungal pathogen *FOL*. The compatibility of this pair of treatments was also evaluated for efficacy and suitability of these two biocontrol agents for use on tomatoes under greenhouse conditions.

## 2 Materials and methods

### 2.1. Collection of plant material and extraction

The fruits of *C*. *colocynthis* were harvested in and around Southern Western Ghats, Tirunelveli District, India during the summer seasons. The collected plants were authenticated and the voucher specimen (Collection number 966 and reference number BSI/CRC/Tech./ 2012–13) of this collection has been submitted to the herbarium of SPKCEES, Manonmaniam Sundaranar University, India. The extraction procedure followed by Visetson *et al* [33] with slight modifications [34]. The yields of fruit extracts (hexane, chloroform, methanol, and water) were 3.93. 2.76, 4.31, and 4. 28g respectively.

### 2.2. Fungal pathogen culture

*FOL* (NFCCI No: 745) was purchased from the National Fungal culture collection of India (NFCCI). Further, the culture was maintained in Potato Dextrose Agar (PDA) medium and incubated at the laboratory condition at 25 ± 3°C. After 48 h, hyphal tips of fungi growing out from the collar region were cut and transferred onto PDA slants [35] and preserved at 24–26°C in the Biological Oxygen Demand (BOD) incubator.

### 2.3. Growth inhibition assay

The Growth inhibition assay was performed by the method of Nene and Thapilyal, [36] with slight adjustments. Two milliliters of fruit extract prepared from each of the solvent extracts at different concentrations (1, 5, and 10%) was mixed with PDA medium. The PDA medium mixed with plant extract was poured into sterilized Petri plates at 15 mL/ plate and allowed to solidify. The plates were then inoculated with the 6 mm discs of a 5-day-old culture of *FOL* grown on PDA and incubated at room temperature (28 ± 3°C). Petri plates were sealed with Parafilm™ and incubated at 27 ± 2°C in a BOD incubator for 6 days. The percent inhibition of radial growth of *FOL* was calculated using Formula 1. Five replicates were used for each treatment and the experiment was repeated three times.


$$\;\text{Growth}\;\text{inhibition}\;\text{\%}=\frac{\text{C}-\text{T}}{\text{C}}\times 100$$ (1)

C = radial growth of *FOL* in the control plate, and T = radial growth of *FOL* with *C*. *Colocynthis* extract.

### 2.4. Analysis of fruit extract through FT-IR and GC-MS

The preliminary antifungal activity was performed with *FOL* with hexane, chloroform, methanol, and water extract of *C*. *colocynthis*. The greatest antifungal activity was observed in methanol and water extract and their chemical constituents were determined with FT-IR and GC-MS analysis.

Perkin Elmer Spectrum One equipped with an ATR-FTIR unit was used to measure the FTIR spectra of the extract in a wavelength range of 450–4000 cm^-1^. The 16 scans were accumulated and averaged. The spectra were collected and analyzed using Spectrum software (Perkin Elmer).

Two hundred microliters of HPLC-grade methanol were dissolved with 200 μl of water extract and examined using a GC-MS spectrometer (Agilent Technologies, Santa Clara, CA). The compound structure, molecular weight, and molecular formula were identified by matching the sample spectrum to those in the database (NIST). The area % was generated using the total ion chromatogram (TIC) peak areas of the eluted compounds.

### 2.5. Maintenance and mass culture of *T*. *viride*

The *T*. *viride* (MTCC no: 800) was purchased from Microbial Type Culture Collection (MTCC). A pure culture of *T*. *viride* was maintained in a PDA medium and maintained at the laboratory condition at 25 ± 2°C.

Sorghum seeds (100 g) were boiled up to 20 to 25 min to soften grains and were then spread out in a laminar airflow chamber to decrease the moisture content. Two grams of calcium carbonate were added (2 g/100 g of seeds) to remove the excess moisture. One-fourth volume of a 250 ml conical flask was filled with these grains (**Fig 4**) and autoclaved at 121°C for 15 min. After cooling, the sorghum seeds were inoculated with a few discs of bio-agent (5 mm) from a 6–7 days old culture of *T*. *viride* conidia grown in PDA medium and incubated at 24 ± 2°C in a BOD incubator for 7–12 days.

### 2.6. Compatibility of *C*. *colocynthis* fruit extract with *T*. *viride*

The compatibility was determined for *T*. *viride* and *C*. *colocynthis* fruit extracts (Methanol and water) by using a PDA medium. The PDA medium was mixed with the *T*. *viride* (2 mL/ 18 mL). Every single milliliter of fungal suspension contained 1 × 10^6^cfu/mL. The medium containing *T*. *viride* was poured into Petri plates and immediately after solidification, two sterile paper discs were placed at equidistance to each other on the seeded medium. Six μL of the 2% fruit extract (Methanol and water) after passing through bacterial filters was added to the first sterile filter paper disc and 6 μL of 0.01 mL of sterile distilled water was added to the second filter paper disc as control, respectively, and incubated at 28 ± 2°C for 5 days. After 5 days, the inhibition zone was observed. The presence of an inhibition zone around the disc indicates incompatibility with fungal isolates and the absence of an inhibition zone indicates compatibility [37].

### 2.7. Efficacy of individual and combination of *T*. *viride* and fruit extract against pathogen

Compatibility result showed methanol extract was compatible with the biocontrol agent *T*. *viride* hence; the methanolic extract was used for further experiments. *T*. *viride* was assayed against *FOL* with the dual-culture method of Rajeev and Mukhopadhyay [38] with little modification. Discs (6 mm diameter) of the pathogen and the antagonist were cut from the edge of the 5-day-old culture and placed on the opposite side of Petri dishes containing PDA, 1 cm away from the edge. Three replications were maintained. Petri dishes were incubated for 4 days at 28 ± 2°C and the mycelial growth of *FOL* was measured. Percent inhibition (PI) of mycelial growth was calculated using the formula suggested by Pandey *et al*. [39].

*C*. *colocynthis* fruit extract was tested individually and in combination with *T*. *viride* against *FOL* by dual culture technique [40]. Sterilized filter paper discs of 3 mm diameter were spotted with 6 μL of *C*. *colocynthis* fruit methanol extract. For combination, 3 μL of *T*. *viride* (2%) and *C*. *colocynthis* methanolic extract (2%) were spotted on sterilized filter paper discs (3 mm diameter) and placed 1 cm away from the edge of the plate containing PDA medium using pour plate technique with the pathogen (dosage of 1 × 10^6^cfu/mL). Four replicates were used for each treatment and the experiment was repeated three times. The plates were incubated at room temperature (28 ± 2°C) for 7-days and the inhibition zone was measured and percent reduction over control was calculated.

### 2.8. Bio formulation

The talc-based formulation of *T*. *viride* was prepared with the adapted procedure of Srivastava *et al*. [41]. After 12 days, the conidia were harvested from the sprouted sorghum seeds and grounded in a laboratory blender to form a suspension. The inoculums were mixed with talcum powder to obtain a conidia concentration of 1 × 10^6^ conidia/ml. Ten grams of carboxy methyl cellulose (CMC) was added to one kg of talc and mixed well. The formulations thus prepared were allowed to dry aseptically overnight (approximately 35% moisture content), then it was packed in sterile polythene bags and stored at 4°C.

In the case of fruit extract, 20 mL of fruit extract was added to 100 g of sorghum seeds. Then, *T*. *viride* was inoculated and incubated for 12 days at 28 ± 2°C and the formulation was prepared as previously described.

### 2.9. Greenhouse study

#### 2.9.1. Effect of bio formulation mixtures on the incidence of wilt disease

Potting medium (red soil: cow dung: vermiculate at 2:1:1, w/w/w) was autoclaved at 105°C for 1 h and filled in pots. Then, the tomato (var.PKM1) seedlings (2–3 true leaf stage) produced in vermiculite were transplanted into autoclaved pot mixture in the surface sterilized (1% mercuric chloride) pots (15 cm diameter, 750 ml volume) at 1 seedling/pot. In all treatments, the talc-based bio formulation mixture was applied as 1:20 (w/w) and foliar spray (20 g/l). Talc-based bio formulations were applied basally at the rate of 10 g/pot. The talc-based bio formulation mixtures were applied 45 days after planting and after 1 day, the plants were inoculated with the spore suspension of *FOL*(1 × 10^6^ conidia/ml). The pathogen alone inoculated served as a control. Soil drenching of 0.1% carbendazim was included as a chemical check (Positive control). Control plants received only the talcum powder with adhesive. Ten days after inoculation, observations on the development of wilt symptoms were made. Three replications (three pots per replication) were maintained and the pots were arranged in a randomized manner.

The treatments of experiment were, T1—Inoculated control, T2 –Un-inoculated control, T3—*T*. *viride*, T4—*C*. *colocynthis*, T5 -*T*. *viride*+ *C*. *colocynthis*, T6—Carbendazim 0.1%.

The measurement of disease index (DI) was determined using the given formula (2) [42].


DI=100×A/B×C (2)

Where A = Sum of the individual scores; B = Total leaves observed; C = Maximum score.

The disease reduction was calculated using the given formula (3).


$$\;\text{Disease}\;\text{suppression}\;\text{efficiency}\;=\frac{\;\text{DI}\;\text{of}\;\text{contol-DI}\;\text{of}\;\text{treatment}\;\text{group}\;}{\;\text{DI}\;\text{of}\;\text{control}\;}\times 100$$ (3)

The disease severity was recorded on a 0–4 scale as described by Song *et al*. [43] where zero represents no infection and four denotes complete infection. The 0–4 scale of the disease severity was classified as follows:

0—No infection. 1—Slight infection, which is about 25% of full scale, one or two leaves turned yellow. 2—Moderate infection, two or three leaves turned yellow, 50% of leaves wilted. 3—Extensive infection, all the leaves turned yellow, 75% of leaves wilted and growth was inhibited. 4—Complete infection, the leaves of the whole plant turned yellow, 100% of leaves wilted, and the plants died.


$$\;\text{Disease}\;\text{incidence}\;\text{(\%)}\;=\frac{\sum \;\text{scale}\;\times \;\text{no}.\text{of}\;\text{plants}\;\text{infected}\;}{\;\text{highest}\;\text{scale}\;\times \;\text{Total}\;\text{no}.\text{of}\;\text{plants}\;}\times 100$$ (4)

The percentage of disease incidence was determined using the formula (4) given by Song *et al*. [43].

#### 2.9.2 Assay of induced defense enzymes

*T*. *viride* and *C*. *colocynthis* fruit extract were used single and in combinations for the induction of defense reactions in tomatoes. The bio formulations treated seeds were sown in earthen pots (size: 0.35 m diameter, 0.50 m height; volume of soil: 0.04 m3) filled with sterilized potting soil at five seeds per pot. Forty-five days after sowing, foliar spray with bio-formulation mixtures was done as described earlier. Bio-formulation-treated plants were divided into two sets. In the first set, treated plants were challenge inoculated with *FOL* (at 1 day after foliar spray) and in the second set, treated plants were not challenged with the pathogen. Plants without prior treatment of bio formulations were inoculated with the pathogen. The plants neither treated with bio formulation nor challenged by the pathogen were kept in control. Three replications were maintained in each treatment; each replicate consisted of five pots and in each pot, one plant was maintained. The experiments were conducted using completely a randomized block design on a greenhouse bench. The humidity in the greenhouse was maintained at around RH 70%. The temperature was adjusted to 26 ± 2°C (day)/20 ± 2°C (night).

Leaf tissues were collected after 5 days of pathogen inoculation. Three plants were sampled from each replication of the treatment separately and were maintained for biochemical analysis. Leaf samples were homogenized with liquid nitrogen in a pre-chilled mortar and pestle. One gram of leaf sample was homogenized with 2 ml of 0.1 M sodium phosphate buffer (pH 7.0) at 4°C. The homogenate was centrifuged for 20 min at 10,000 rpm. The supernatant was used as a crude enzyme extract for assaying PO [44] and polyphenol oxidase (PPO) [45]. An enzyme extracted in 0.1 M sodium citrate buffer (pH 5.0) was used for the estimation of chitinase [46] and β-1,3-glucanase.

### 2.10. Statistical analysis

The data on the effect of the treatments on the growth of pathogens, severity of diseases, and activity of enzymes in rice plants were analyzed with analysis of variance (ANOVA), and treatment means were compared by Tukey’s-family error test (P < 0.05) for pairwise comparison using Minitab^®^16 software package. The data on *in vitro* bacterial growth inhibition, disease incidence, disease index, and suppression efficiency were arcsine transformed before undergoing statistical analysis.

## 3. Results

### 3.1. Growth inhibition assay

Fruit extracts of *C*. *colocynthis* using hexane, chloroform, methanol, and water at various concentrations (2.5, 5, and 10%) were evaluated for antimicrobial activity against the pathogen *FOL*. All the extracts at 10% concentration were effective in inhibiting the radial growth of *FOL*, compared to the control. The methanolic fruit extract of *C*. *colocynthis* at 10% was most effective in inhibiting the mycelial growth of *FOL* (12.32 mm) lowed by 10% of water, chloroform, and hexane with 23.61, 35.62, and 40.48 mm respectively. In contrast, *FOL* at control had mycelial growth of 90.0 mm (Table 1). All the treated concentration of *C*. *colocynthis* was significantly different from the control (*F*_11,28_ = 39.26, *P* ≤ 0.001).

**Table 1:** Effect of *C*. *colocynthis* fruit extract on the mycelial growth of *FOL*. Values are mean of three replications. Means (±SEM) followed by the same letter within columns indicate no significant difference (*P* ≤ 0.05) in a Tukey’s test.

*C*. *colocynthis* extract	Treatment concentration(%)	Mycelial growth (mm)	Reduction over control (%)
Hexane	2.5	73.73^B^	18.07
	5	60.02^C^	33.31
	10	40.48^FG^	55.02
Chloroform	2.5	60.31^C^	32.98
	5	48.52^DE^	46.08
	10	35.62^GH^	60.42
Methanol	2.5	43.56^EF^	51.60
	5	32.21^H^	64.21
	10	12.32^J^	86.31
Water	2.5	52.23^D^	41.96
	5	45.45^EF^	49.50
	10	23.61^I^	73.76
Control	-	90.0^A^	-

### 3.2. FT-IR and GC-MS analysis

**Fig 1:**
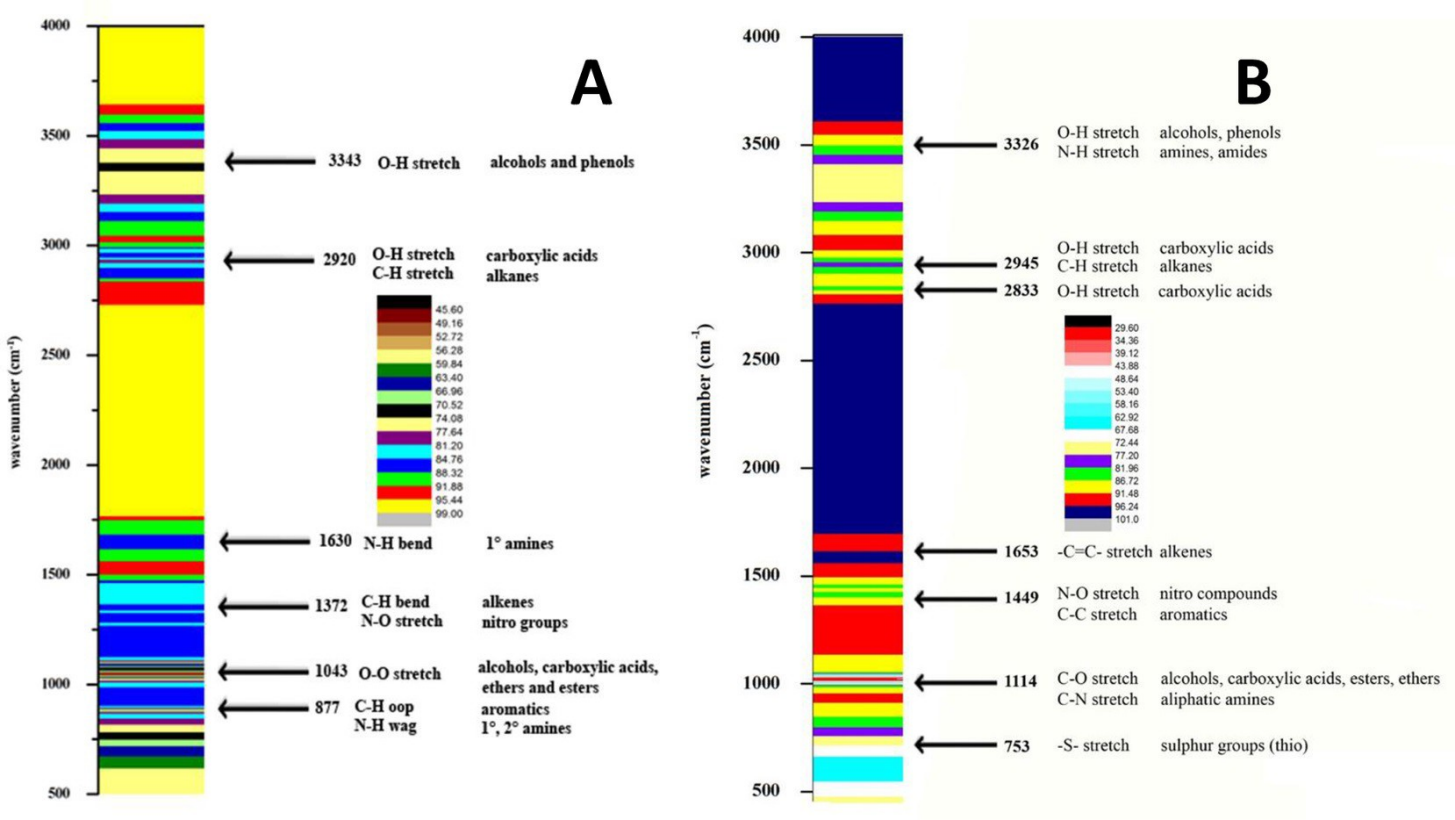
(A) FT-IR spectrum of *C*. *colocynthis* methanolic extract and (B) FT-IR spectrum of *C*. *colocynthis* water extract.

The FT-IR spectrum of methanolic extract (**Fig 1A**) shows the presence of several bands corresponding to the wave numbers 3343, 2920, 1630, 1372, 1043, and 877 cm^-1^. The band detected at 3343 cm^-1^ arises due to the O-H stretch of alcohols and phenols. The band observed at 2920 cm^-1^ formed due to the O-H stretch of carboxylic acids and also due to the C-H stretch of alkanes. The N-H bend of primary amines was found at 1630 cm^-1^. The bands arise at 1372 cm^-1^ attributed to the C-H bend of alkenes and the stretch of N-O due to the nitro groups. The carboxylic acids, alcohols, esters, and ethers have O-O stretch at 1043 cm^-1^. The out-of-plane (oop) stretch of aromatics (C-H) was found at 877 cm^-1^ and also the N-H wag of primary and secondary amines was found in this extract.

Similarly, the FT-IR spectrum of water extract (**Fig 1B**) exhibits major peaks consistent with the wavenumber 3326, 2945, 2833, 1653, 1449, 1114, and 753 cm ^-1^. The broad band at 3326 cm^-1^ arises due to the O-H stretch and N-H stretch of alcohols and phenols, amides, and amines respectively. The band observed at 2945 cm^-1^ formed due to the O-H stretch of carboxylic acids and also due to the C-H stretch of alkanes. The O-H stretch of carboxylic acids was found at 2833 cm^-1^. The band found at 1653 cm^-1^ attributes the–C = C- stretch of alkenes. The nitro compounds have an N-O stretch and aromatics have a C-C stretch at 1449 cm^-1^. The stretching of alcohols, carboxylic acids, esters, ethers (C-O), and aliphatic amines (C-N) was found at 1114 cm-1. The absorbance band at 753 cm-1 indicates the presence of Sulphur groups.

**Fig 2:**
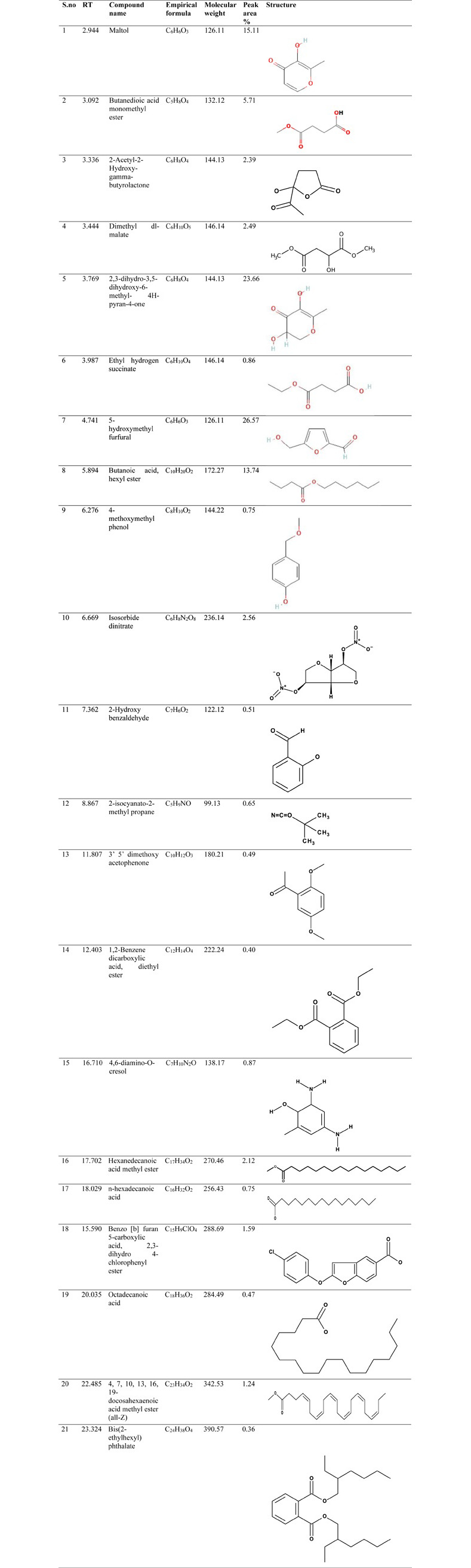
Chemical characterization of *C*. *colocynthis* methanolic extract through GC-MS analysis.

**Fig 3:**
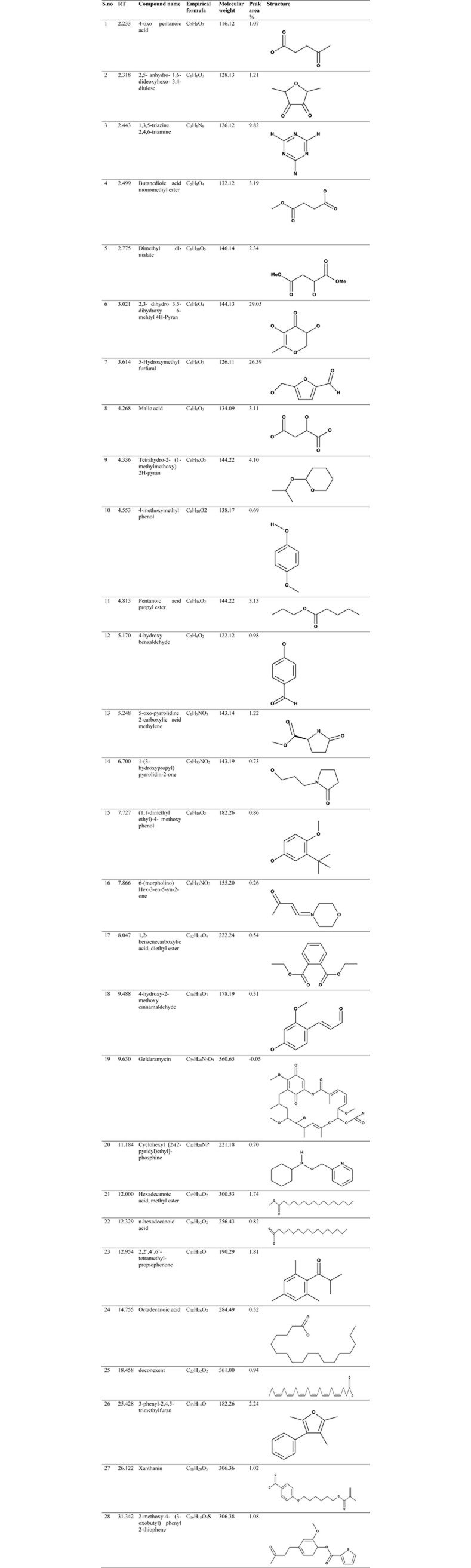
Chemical characterization of *C*. *colocynthis* water extract through GC-MS analysis.

The compounds present in methanolic and water extract of *C*. *colocynthis* were identified and the mass spectrum of the analysis was confirmed with comparisons to the NIST library. The retention time (RT), compound name, molecular weight (g/mol), molecular formula, peak area percentages, and chemical structures were presented in Figs 2 and 3.

**Fig 4:**
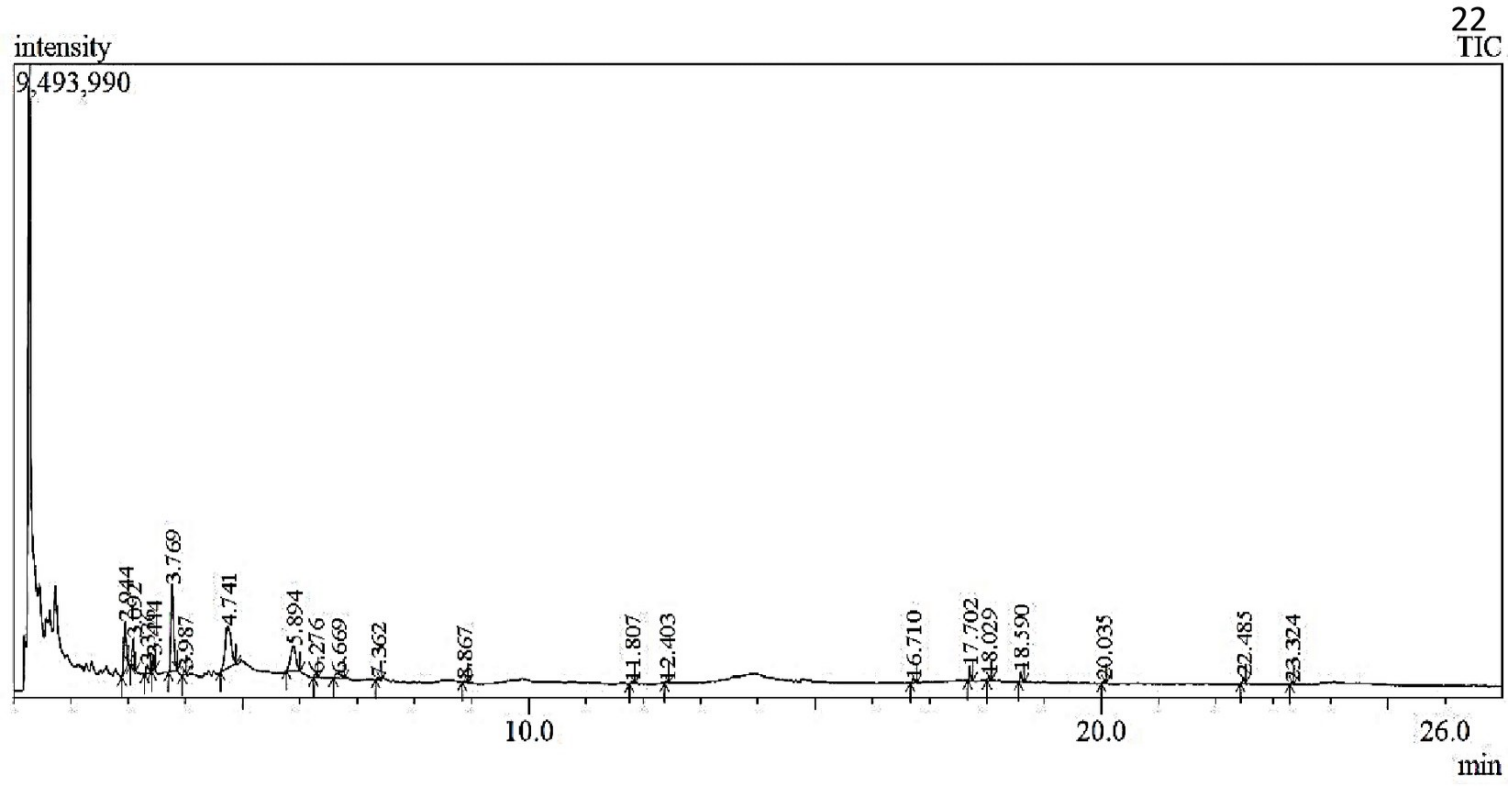
GC-MS analysis of *C*. *colocynthis* methanolic extract.

**Fig 5:**
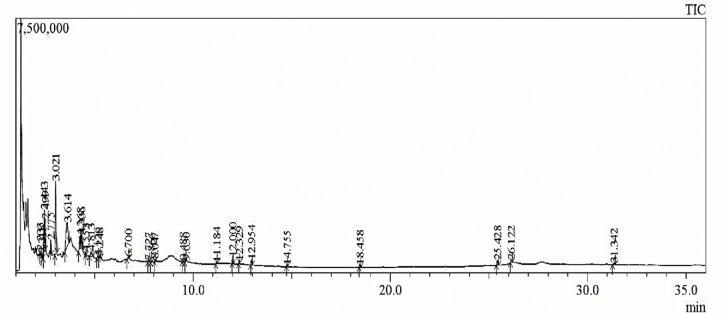
GC-MS analysis of *C*. *colocynthis* water extract.

The GC-MS spectrum of methanolic extract (Fig 2) revealed that 21 major compounds were presented in **Fig 4**. The methanolic extract displayed 5-hydroxymethyl furfural (26.57%), 2,3-dihydro-3,5- dihydroxy-6-methyl- 4H-pyran-4-one (23.66%), Maltol (15.11%) and Butanoic acid, hexyl ester (13.74%) with higher peak area percentage. On the other hand, water extract shows 28 chief compounds (**Fig 5**) with significant matches to 2,3- dihydro 3,5- dihydroxy 6-methyl 4H-Pyran (29.05%), 5-Hydroxymethyl furfural (26.39%) and 1,3,5-triazine 2,4,6-triamine (9.82%).

### 3.3. Compatibility of *C*. *colocynthis* fruit extract with *T*. *viride*

The antagonist fungi *T*. *viride* and *C*. *colocynthis* methanolic extract were tested for their compatibility *in vitro*. The absence of an inhibition zone around the paper disk was observed in *C*. *colocynthis* methanolic extract. This result indicated that the biocontrol agent *T*. *viride* was compatible with *C*. *colocynthis* methanolic extract. On the other hand, *C*. *colocynthis* water extract was not compatible with the biocontrol agent, showing an inhibitory effect on *T*. *viride*.

### 3.4. *In vitro* screening of fungal antagonists and *C*. *colocynthis* against FOL

**Fig 6:**
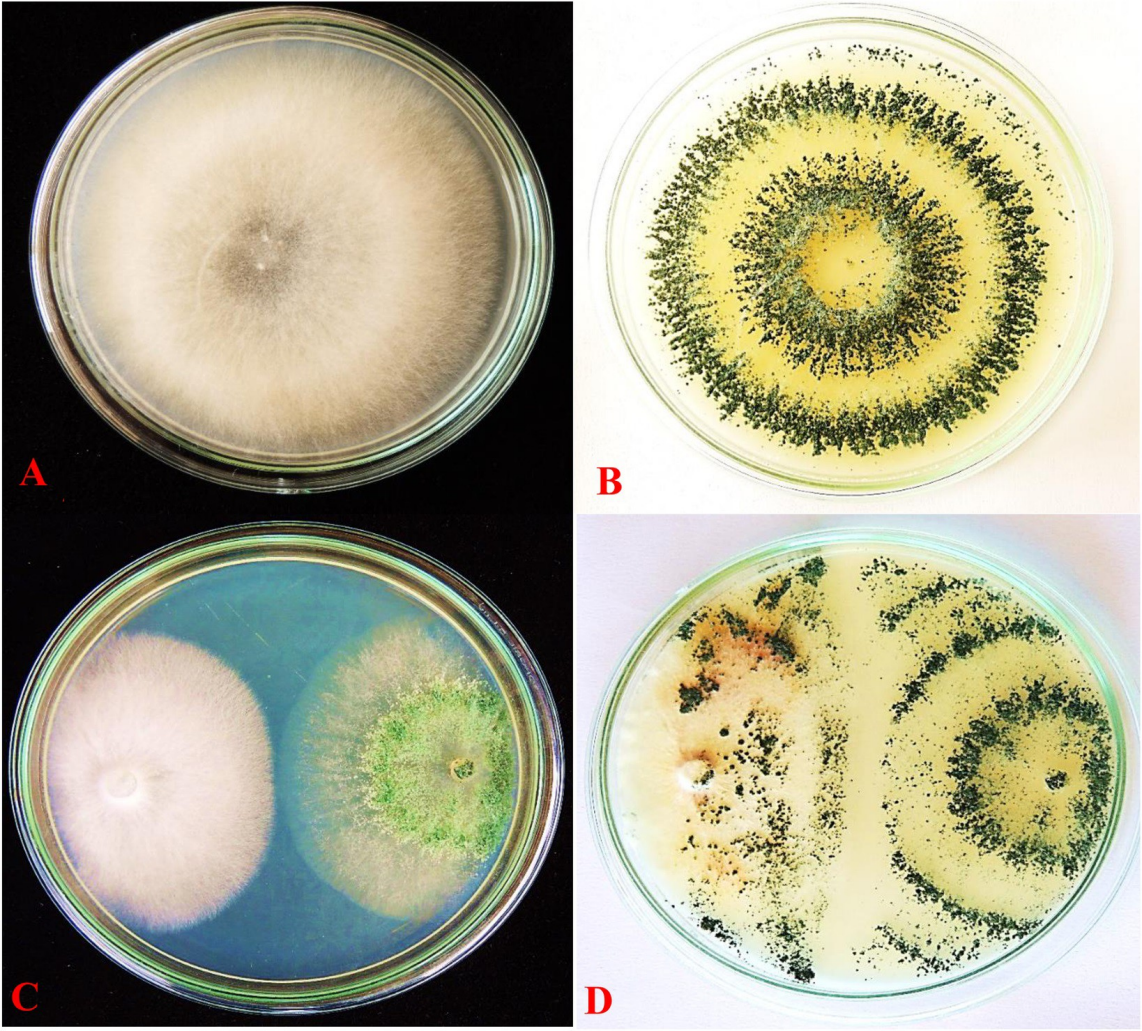
*In vitro* screening of fungal antagonists and *C*. *colocynthis* against FOL (A) FOL control (B) *T*. *viride* control (C) Radial growth of T. viride and FOL, (D) Mycoparasitic action of *T*. *viride* on the mycelium of FOL.

Treatments with *T*. *viride* and *C*. *colocynthis* methanolic extract were tested individually and in combination to assess the radial growth of *FOL*. Compared to the control plates (**Fig 6A and 6B**) all the treatments were effective in reducing the mycelial growth of the pathogen *FOL*. The dual culture results show that *T*. *viride* alone reduced the mycelial growth of *FOL* by 26 mm (**Fig 6C**). After 9 days of *In-vitro* screening of *T*. *viride* were observed against the wilt pathogen *FOL* (**Fig 6D**). However, the combined application of *T*. *viride* and *C*. *colocynthis* methanolic extract resulted in the highest inhibition of 2.5 mm with least mycelial growth of 14.21 mm and accounting for 82.92% reduction of *FOL* mycelial growth compared to control. The control plates recorded the highest mycelial growth of 83.24 mm (Table 2). All the treatment concentrations were significantly different from the control (*F*_4,10_ = 34.59, *P* ≤ 0.001).

**Table 2:** Effect of *T*. *viride* and *C*. *colocynthis* fruit extract on the mycelial growth of *FOL*.

S.no	Treatments	Mycelial growth (mm)	Reduction over control (%)	Inhibition zone (mm)
**1.**	*T*. *viride*	26.25±0.93^cd^	68.46	1.8±0.07^b^
**2.**	*C*. *colocynthis* 2%	41.53±0.23^b^	50.10	1.4±0.07^b^
**2.**	*T*. *viride* + *C*. *colocynthis* 2%	14.21±0.31^d^	82.92	2.5±0.09^a^
**3.**	Carbendazim (0.1%)	28.42±0.54^c^	65.85	1.6±0.51^b^
**4.**	Control	83.24±0.21^a^	-	-

### 3.5. Effect of bio formulation mixtures on the incidence of wilt disease

**Fig 7:**
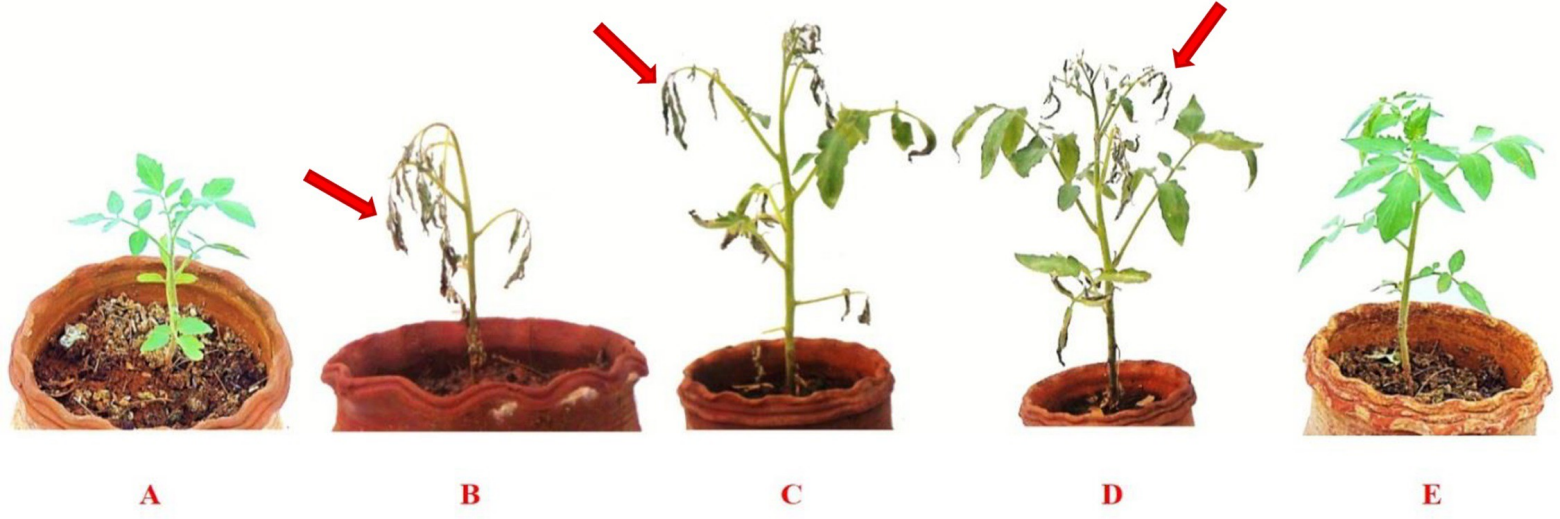
Effect of bio formulation mixtures on the incidence of wilt disease (A) Positive control (0.1% carbendazim treatment), (B) *FOL* alone inoculated, (C) *C*. *colocynthis* extract + *FOL*, (D) *T*. *viride* + *FOL*, (E) *C*. *colocynthis* extract + *T*. *viride* + *FOL*. Arrow mark indicate the wilt symptoms.

Talc-based formulations of *T*. *viride* and *C*. *colocynthis* methanolic extract were tested individually or in combination, for their efficacy against *FOL* under pot culture conditions. The biocontrol agents and *C*. *colocynthis* methanolic extract significantly reduced the wilt disease by 47.23 and 41.05% compared to the control (Table 3). Noticeably, a combination of *T*. *viride* and *C*. *colocynthis* methanolic extract significantly lower the disease incidence (**Fig 7**) by 21.92%. Whereas, carbendazim at 0.1% decreases the disease incidence by 32.53% only. All the treatment concentrations were significantly different from to control (*F*_4,10_ = 30.29, *P* ≤ 0.001). However, *C*. *colocynthis* methanolic extract was not significant to *T*. *viride* (*F*_4,10_ = 30.29, *P* ≤ 0.201) and carbendazim (*F*_4,10_ = 30.29, *P* ≤ 0.052).

**Table 3:** Efficacy of *T*. *viride* and *C*. *colocynthis* fruit extract in control of wilt disease. Means (±SEM) followed by the same letter within columns indicate no significant difference (*P* ≤ 0.05) in a Tukey’s test.

S.no	Treatments	*FOL*	Disease incidence (%)	Disease Index (%)	Reduction over control (%)
1.	*T*. *viride*	+	47.23b	52.71b	28.95
3.	*C*. *colocynthis* 2%	+	41.05bc	45.43c	44.03
5.	*T*. *viride + C*. *colocynthis* 2%	+	21.92d	27.02d	66.78
6.	Carbendazim (0.1%)	+	32.53c	41.23c	49.32
7.	Control	+	85.24a	81.36a	-

The disease index in control was 81.36% and decreased to 52.71, 45.43 and 27.02% with the individual treatment of *T*. *viride* and *C*. *colocynthis* methanolic extract and in combination respectively. The result revealed that all the treatments were significant difference compared to the control (*F*_4,10_ = 36.24, *P* ≤ 0.001). But, *C*. *colocynthis* methanolic extract was not significant to *T*. *viride* (*F*_4,10_ = 36.24, *P* ≤ 0.108). Interestingly, a combination of antagonistic fungi and *C*. *colocynthis* methanolic extract resulted in a prominent upsurge in the percentage of disease reduction over control (66.78%) than the chemical carbendazim (49.32%).

### 3.6. Assay of induced defense enzymes

Defense-related enzymes viz., peroxidase, polyphenol oxidase, β-1,3-glucanase, and chitinase showed higher activity in all treatments compared with non-inoculated control.

**Fig 8:**
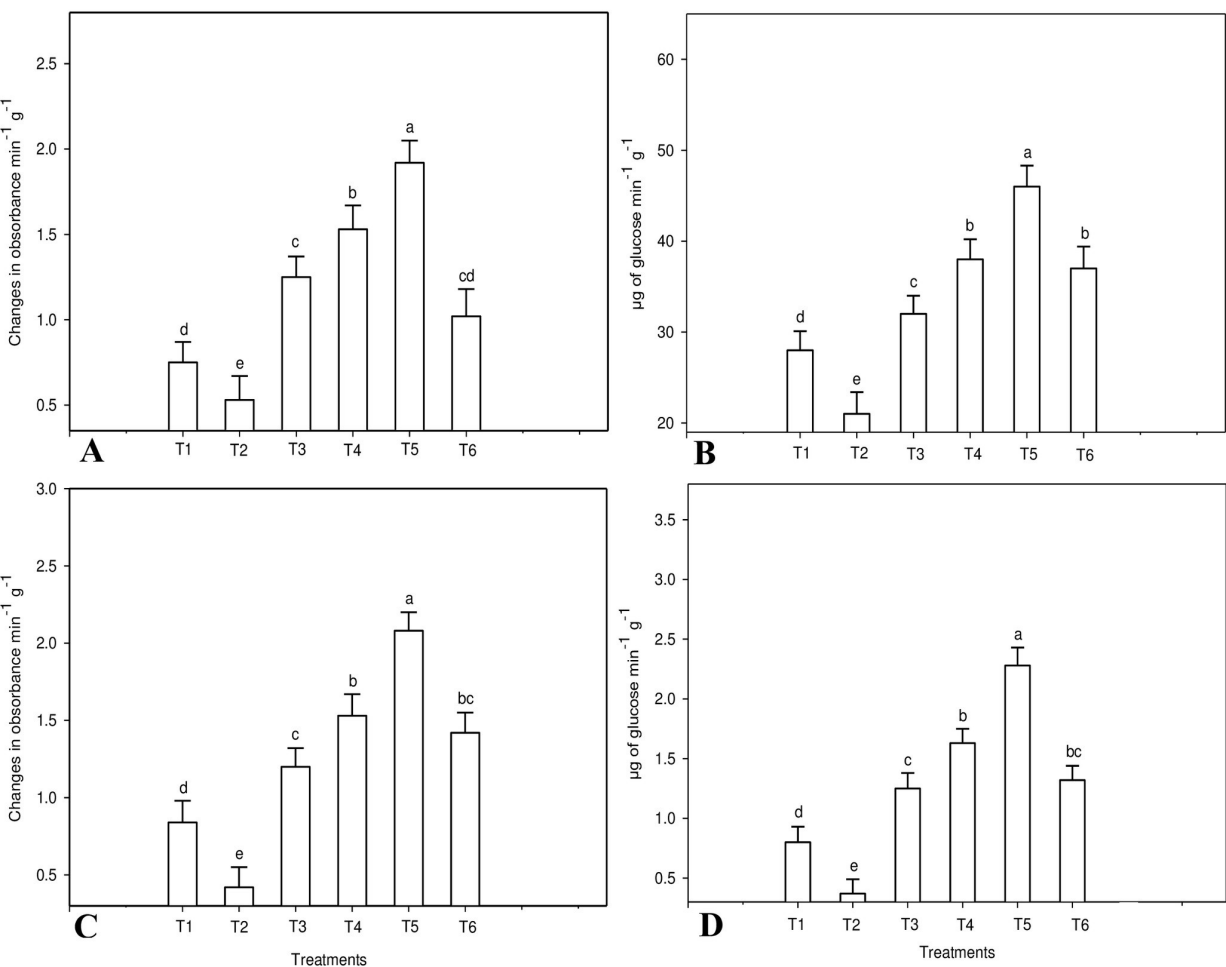
Defense related enzyme activity of: (A) peroxidase; (B) polyphenol oxidase (C) β-1,3-glucanase; and (D) chitinase. were, T1—Inoculated control, T2 –Un-inoculated control, T3—T. viride, T4—C. colocynthis, T5 -T. viride+ C. colocynthis, T6—Carbendazim 0.1%. Means (± (SE) standard error) followed by the same letters above bars indicate no significant difference (P≤0.05) according to a Tukey test.

Peroxidase enzyme activity was strongly induced in the combined treatment of *T*. *viride* and *C*. *colocynthis* methanolic extract followed by the treatment of *C*. *colocynthis* extract, *T*. *viride*, carbendazim and inoculated control (**Fig 8A**). Peroxidase enzyme activity in non-inoculated control (*F*_5,12_ = 21.31, *P* ≤ 0.001) was significantly different than all the treatments. However, the treatment of carbendazim did not yield significant results compared to the treatment of *T*. *viride* (*F*_5,12_ = 21.31, *P* ≤ 0.063) and inoculated control (*F*_5,12_ = 21.31, *P* ≤ 0.058).

Similarly, the polyphenol oxidase enzyme was also increased in the combined treatment of *T*. *viride* and *C*. *colocynthis* methanolic extract (**Fig 8B**). Polyphenol oxidase enzymes induced in non-treated control plants are considered to be statistically significant when compared to other treatments (*F*_5,12_ = 27.87, *P* ≤ 0.001). Pathogen-inoculated plants were gradually increased in PPO with 2.08, 1.53, 1.42, and 1.2 absorbance/min/g by the treatment of a combination of *T*. *viride* and *C*. *colocynthis* methanolic extract, *C*. *colocynthis* extract, carbendazim, and *T*. *viride*.

As found in PO and PPO, an increase in β-1,3-glucanase enzyme activity was observed in all the treatment concentrations. β-1,3-glucanase enzyme activity in pathogen non-inoculated control was 21 μg min^-1^ g^-1^ and increased to 28, 32, 37. 38 and 46 μg min^-1^ g^-1^ with the treatment of pathogen inoculated control, *T*. *viride*, carbendazim, *C*. *colocynthis*, and a combination of *T*. *viride* and *C*. *colocynthis* methanolic extract (**Fig 8C**). The results revealed that all the treatments increased the β-1,3-glucanase enzyme activity and it illustrates a significant difference compared to the control (*F*_5,12_ = 26.33, *P* ≤ 0.001). Furthermore, the treatment of *C*. *colocynthis* and carbendazim (*F*_5,12_ = 26.33, *P* ≤ 0.952) was not significant to each other.

Results obtained from chitinase activity observed that all the treatment concentrations triggered the chitinase activity. The result demonstrates that combined treatment of *T*. *viride* and *C*. *colocynthis* methanolic extract shows an increased in chitinase activity (**Fig 8D**) at 2.28 μg min^-1^ g^-1^. Plants exposed with the treatment of inoculated control, *T*. *viride*, carbendazim, and *C*. *colocynthis*, whose chitinase activity was increased to 0.8, 1.25, 1.32, and 1.43 μg min^-1^ g^-1^ respectively. Chitinase activity in all the treatments shows significantly different judge against its non-inoculated control (*F*_5,12_ = 24.05, *P* ≤ 0.001). But, the chitinase activity of carbendazim was not significant in the treatment of *C*. *colocynthis* methanolic extract (*F*_5,12_ = 24.05, *P* ≤ 0.362) and *T*. *viride* (*F*_5,12_ = 24.05, *P* ≤ 0.771).

## 4 Discussion

Biological control agents developed for agricultural crop pathogens, especially soil-borne plant pathogens from botanicals, in combination with micro-organisms are gaining momentum as a viable system in controlling plant pathogen diseases with more native and environmentally acceptable alternatives to the current heavy dependence upon harmless chemical treatments.

Controlling crop diseases by application of plant products was previously suggested by many researchers [47]. In the current study, we assessed the antifungal activity of *C*. *colocynthis* fruit extracts (Hexane, chloroform, methanol, and water) against a vascular wilt pathogen. Methanol and water extracts were effective in reducing the mycelial growth of *FOL*. Methanol extract of *C*. *colocynthis* controlled the *FOL* by 44%. Many researchers have described the antifungal activity of different botanicals against phytopathogenic fungi *FOL* [48,49].

Crop Survey report of 2012, reviewed *F*. *oxysporum* ranking as the third most destructive plant pathogen [50]. It causes severe yield losses of both field and greenhouse tomato crops [11]. Treatments to control Fusarium wilt in tomato crops have become one of the most challenging tasks in terms of management [51].

On the other hand, plant exposure to beneficial microbes also induced systemic resistance [52]. The mycoparasitic soil fungi, *T*. *viride*, is the most extensively used biocontrol agent and it plays an important role in mycoparasitism, antibiosis, and production of elicitors in plants [53]. In this study use of the antagonistic fungi, *T*. *viride* inhibits the pathogen *FOL* by 68%. Similar results by John *et al*. [54] and Singh and Kumar [48], reported that *T*. *viride* effectively controlled the fungal pathogen *FOL*. When combined the fruit extract from *C*. *colocynthis* and *T*. *viride* resulted in effective *FOL* control. The combination of the two was also compatible. Muthu Kumar *et al*. [55] reported that *T*. *viride* was also compatible with the extract of *Allium sativum × Allium cepa*.

An essential principle for a successful application of *T*. *viride* is the preparation of microbial biomass with high effectiveness and an inexpensive ingredient. The advances in biological control for crops is being contingent not only on the antagonist effectiveness of biocontrol agents but also on the costs of mass production. In this report, low-cost sorghum seeds were shown to be a suitable and affordable substrate to support *T*. *viride* growth for mass production. Thangavelu *et al*. [56], described five different organic substrates (rice bran, rice chaffy grain, farmyard manure, banana pseudo stem, and dried banana leaf) that could also be used as a substrate for the growth of *Trichoderma* sp, demonstrating that this technology is within reach of farmers in most countries. In parallel, Srivastava *et al*, (2010) [41] used six different substrates (Jhangora, mandua, sorghum, polygonum, rice straw, wheat straw) for mass cultivation of *Trichoderma* sp. showing higher biomass production in sorghum seeds next to Jhangora and mandua. Our results show that *T*. *viride* requires a minimum period of 7–12 days to grow on the substrate (Sorghum seeds). Thus, strongly suggests that it is possible to use sorghum seeds as an inexpensive substrate to produce and harvest *T*. *viride* for use in agriculture fields to suppress phytopathogens.

Our results show that tomato plants treated with *T*. *viride* display 29% protection against *FOL*. Soil application of the biocontrol agent *T*. *viride* successfully reduces soil-borne pathogens [57,58]. Dubey *et al*. [59] reported that *Trichoderma* sp viz. *T*. *viride*, *T*. *harzianum*, and *T*. *virens* inhibit the mycelial growth of *FOL*. Significantly improved disease protection of 67% against *FOL* was observed only in plants treated with both *C*. *colocynthis* extract and *T*. *viride*. Similar reports have been published that support successful biocontrol agents used in combination which were significantly better than a single agent [41,55,58,60].

Priming for enhanced defense is a common feature of induced resistance and can explain the broad-spectrum efficiency that is typical for many induced resistance phenomena. In this current study, tomato seedlings treated with *C*. *colocynthis* extract and *T*. *viride* were evident from the increased activity of defense-related enzymes. The defense-related enzymes viz., PO, PPO, chitinase, and β-1,3 glucanase stimulate induced systemic resistance to protect themselves against pathogen attack [4, 61]. The induction of defense-related enzymes is a sign of induced systemic resistance in plants. Defense-related enzymes like PO and PPO have been shown to have direct antifungal activities [62]. Plants PO are generally secreted into cell walls and vacuoles or the surrounding medium [63,64]. Moreover, PO was described to play a part in the production of reactive oxygen species, which are active in the signal transduction pathway of plant defense mechanism [65].

Production of PO (1.92 absorbance/min/g), PPO (2.08 absorbance/min/g), β-1,3 glucanase (46 μg/min/g), and chitinase (2.28 46 μg/min/g) was significantly higher in tomato plant treated with *C*. *colocynthis* extract and *T*. *viride*, as compared to un-inoculated control. Chen *et al*. [66] observed that PO and PPO activity was enhanced in the biocontrol of cucumber Fusarium wilt by the treatment of *Bacillus subtilis* B579. Hence, our study supports the hypothesis that the reductions in disease index in tomato plants treated with fungal pathogen *FOL* are due to the induced systemic resistance which suppresses the *FOL* by the production of defense-related enzymes.

## 5 Conclusion

As an endnote, our study showed that a combination of methanolic fruit extract of *C*. *colocynthis* and *T*. *viride* reduced Fusarium wilt disease significantly better than either a single application of *C*. *colocynthis* or *T*. *viride*, alone. This was due most likely to induced defense enzyme activity. Induction of resistance described in the current study is an easy and less expensive technique for procuring maximum protection in fungal pathogen disease management.

## Supporting information

S1 File All the supporting information including Fruit of C. colocynthis, Mass culture of T. viride using sorgum seeds and growth rate of plant (A) Control plant (B) T. viride inoculated plant was displayed (S1 Fig 1–3). Also, the growth rate comparison of T. viride and F. oxysporum. Means (±(SE) standard error) indicate no significant difference (P≤0.05) according to a Tukey test was displayed (S1 Table).
(DOCX)

S1 Graphical abstract (DOCX)

S1 Raw data (XLSX)
