# Supplementary material for: Systematic induced resistance in Solanum lycopersicum (L.) against vascular wilt pathogen (Fusarium oxysporum f. sp. lycopersici) by Citrullus colocynthis and Trichoderma viride
Source: PLoS One. 2023 May 2;18(5):e0278616. doi: 10.1371/journal.pone.0278616 (PMC10153711; doi:10.1371/journal.pone.0278616)
Supplement: S1 File — All the supporting information including Fruit of C. colocynthis, Mass culture of T. viride using sorgum seeds and growth rate of plant (A) Control plant (B) T. viride inoculated plant was displayed (S1 Fig 1–3). Also, the growth rate comparison of T. viride and F. oxysporum. Means (±(SE) standard error) indicate no significant difference (P≤0.05) according to a Tukey test was displayed (S1 Table). (DOCX) [file pone.0278616.s001.docx]

**Supplementary Files**

S1 Fig.


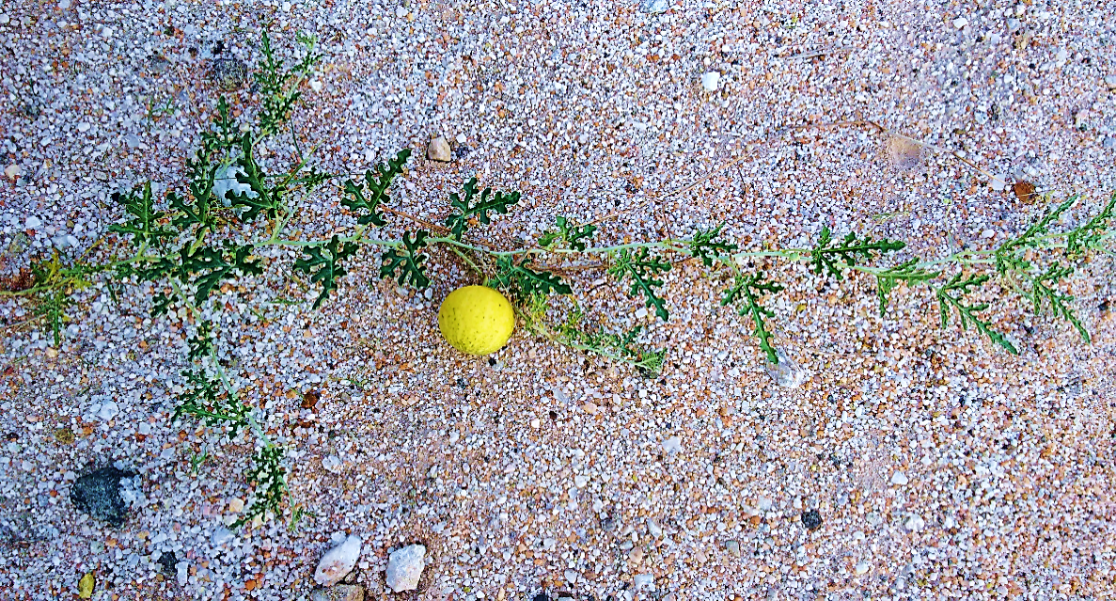

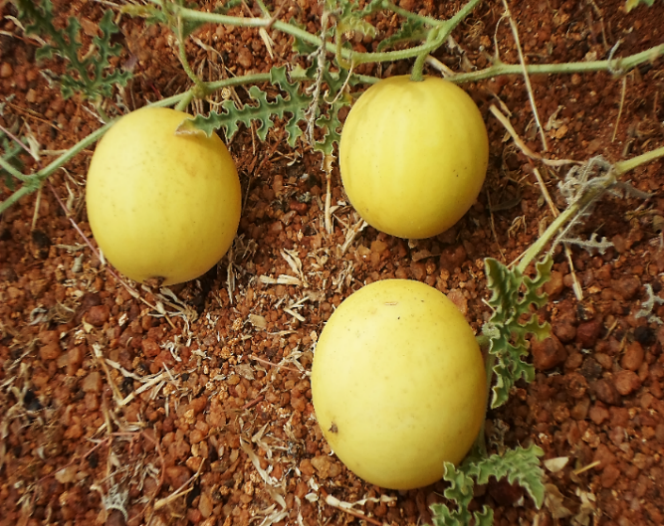

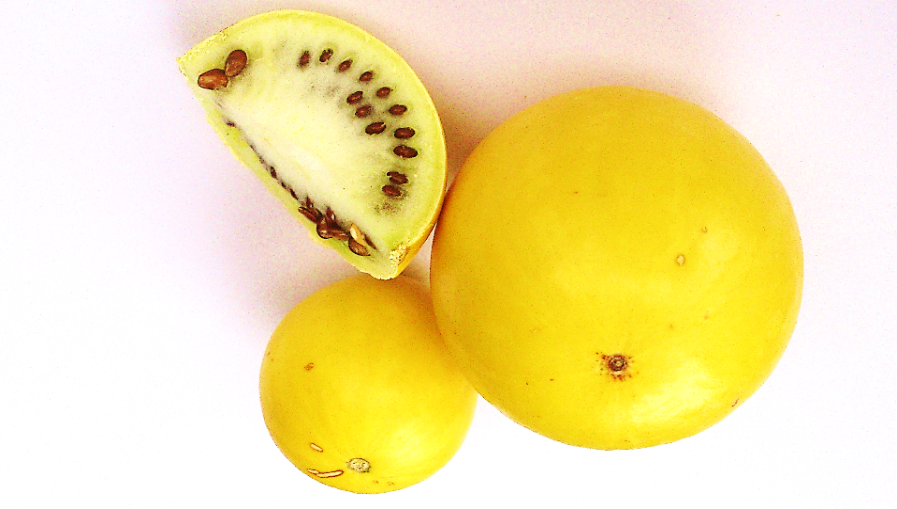


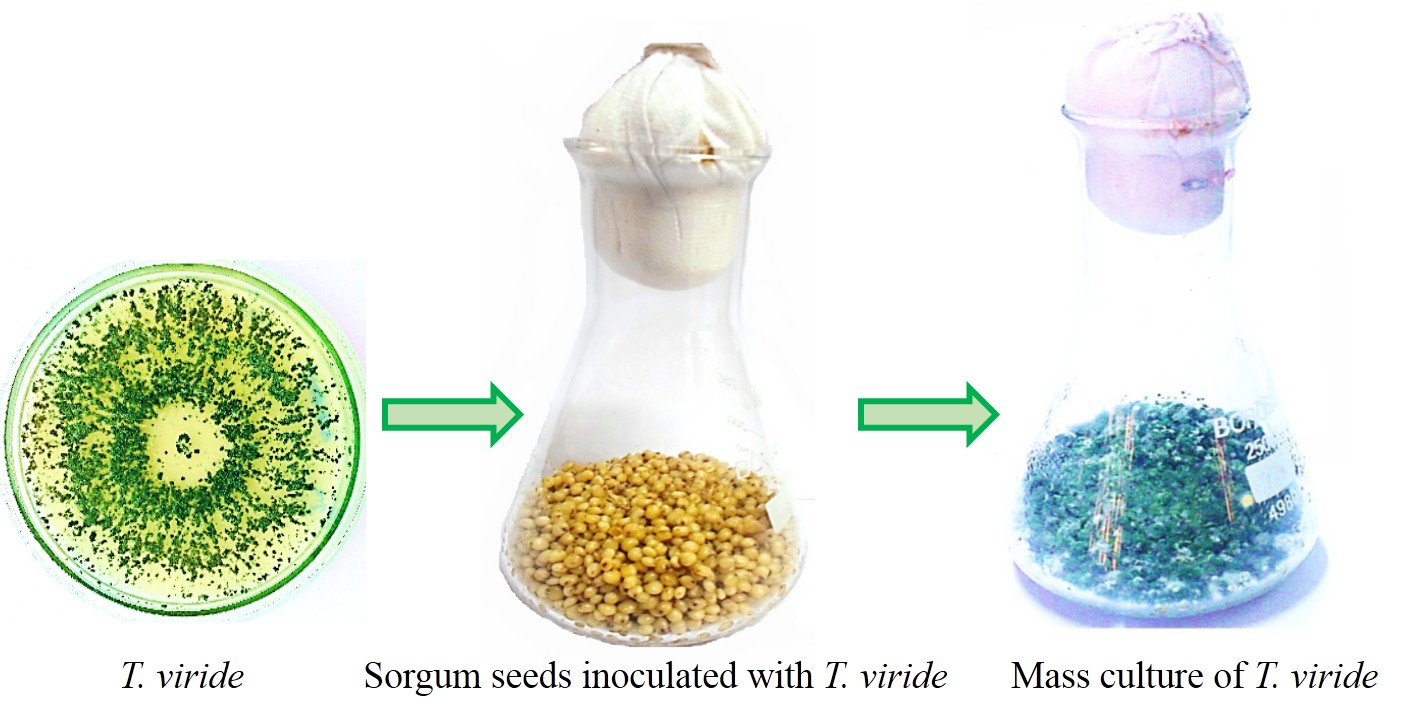
S2 Fig.

**S3 Fig.**


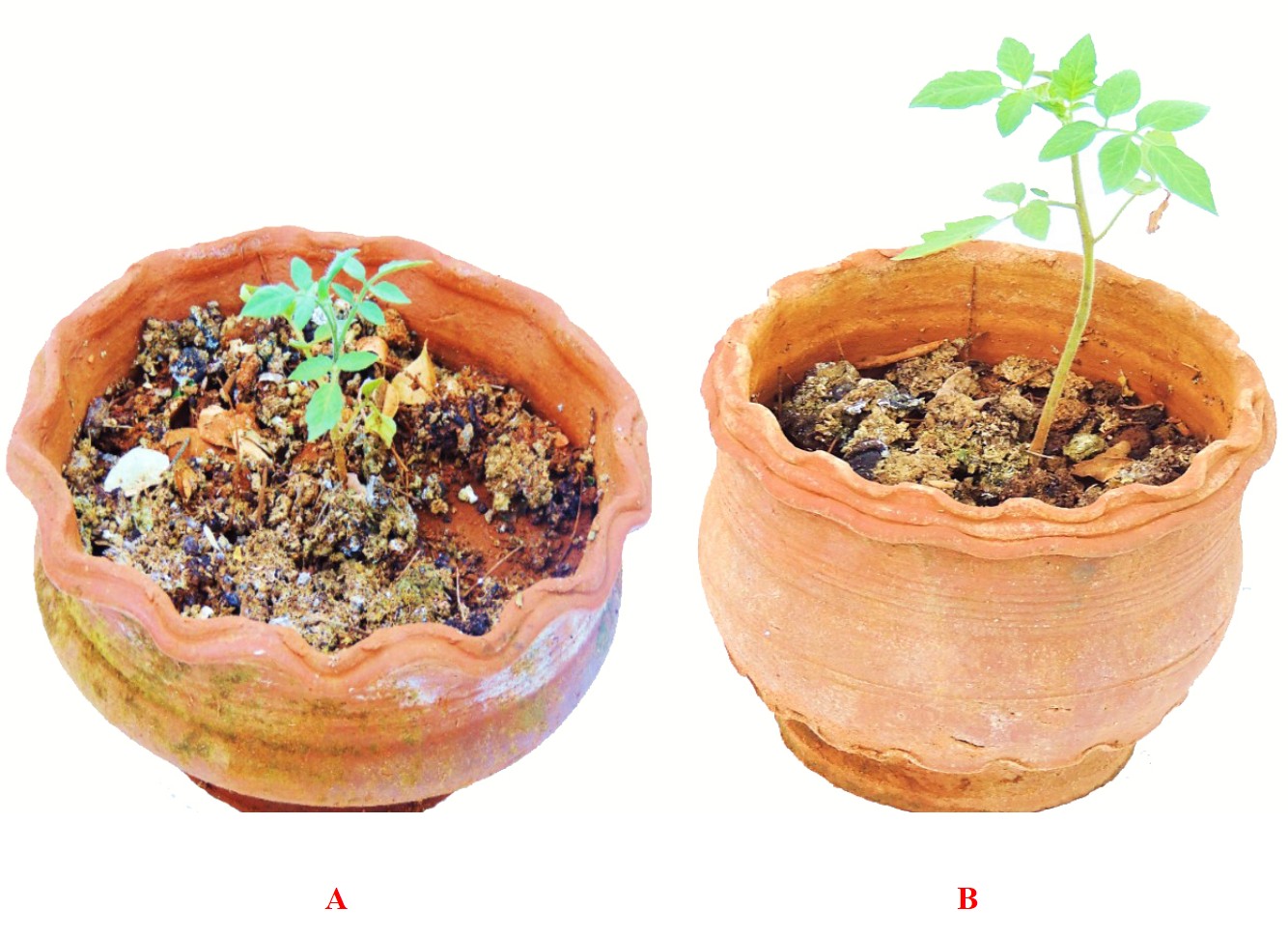


**S1 Table**. Growth rate comparison of *T. viride* and *F. oxysporum*. Means (±(SE) standard error) indicate no significant difference (P≤0.05) according to a Tukey test.

| S.no | Time (hours) | Growth of *T. viride*  radius (mm) | Growth of *F. oxysporum*  radius (mm) | Growth rate ratio |
| --- | --- | --- | --- | --- |
| 1. | 0 | 0 | 0 | 0 |
| 2. | 24 | 6.3±1.79 | 3.7±1.13 | 1.70 |
| 3. | 48 | 19.2±1.32 | 6.3±2.72 | 3.04 |
| 4. | 72 | 37.6±2.34 | 11.2±1.62 | 3.35 |
| 5. | 96 | On the walls of Petri dish 44.8 | 15.4±2.13 | 2.90 |
| 6. | 120 | Overwhelming growth | 20.7±1.83 | - |
