# Supplementary figures and images for: Systematic induced resistance in Solanum lycopersicum (L.) against vascular wilt pathogen (Fusarium oxysporum f. sp. lycopersici) by Citrullus colocynthis and Trichoderma viride
Source: PLoS One. 2023 May 2;18(5):e0278616. doi: 10.1371/journal.pone.0278616 (PMC10153711; doi:10.1371/journal.pone.0278616)

GRAPHICAL ABSTRACT


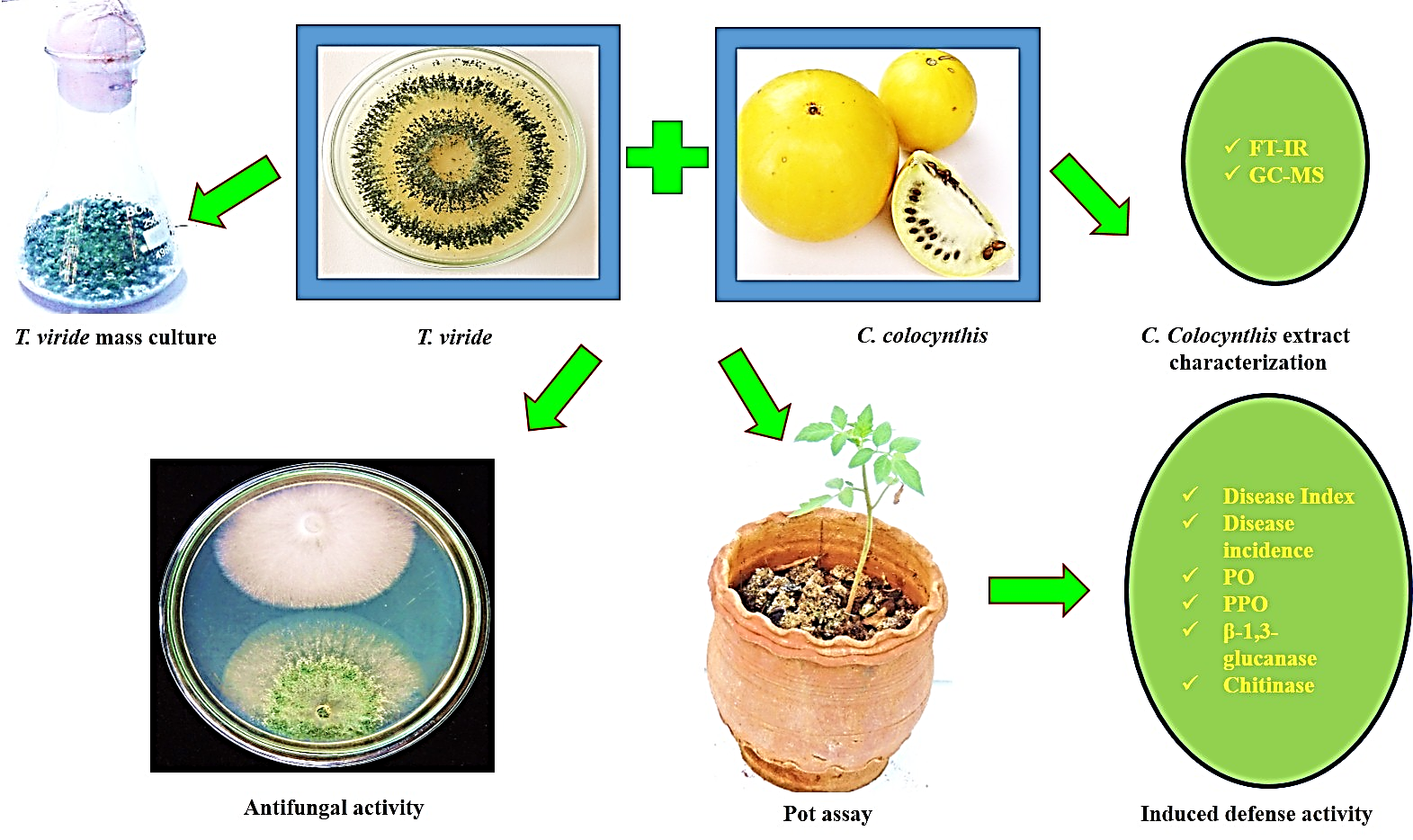

Supplement: S1 Graphical abstract — (DOCX) [file pone.0278616.s002.docx]
